# Continuing Medical Education in the Post COVID-19 Pandemic Era

**DOI:** 10.2196/49825

**Published:** 2023-11-15

**Authors:** Debra Blomberg, Christopher Stephenson, Teresa Atkinson, Anissa Blanshan, Daniel Cabrera, John T Ratelle, Arya B Mohabbat

**Affiliations:** 1 General Internal Medicine Mayo Clinic Rochester, MN United States; 2 Department of Cardiovascular Disease Mayo Clinic Rochester, MN United States; 3 Marketing Mayo Clinic Rochester, MN United States; 4 School of Continuous Professional Development Mayo Clinic Rochester, MN United States; 5 Department of Hospital Internal Medicine Mayo Clinic Rochester, MN United States

**Keywords:** continuing medical education, post COVID-19 pandemic, content development, collaboration, audience, marketing, budgeting, accreditation, evaluation and outcomes, competitive assessment, education, development, assessment, continuing education, medical education, framework

## Abstract

Continuing medical education (CME) is a requirement for medical professionals to stay current in their ever-changing fields. The recent significant changes that have occurred due to the COVID-19 pandemic have significantly impacted the process of providing and obtaining CME. In this paper, an updated approach to successfully creating and administering CME is offered. Recommendations regarding various aspects of CME development are covered, including competitive assessment, marketing, budgeting, property sourcing, program development, and speaker and topic selection. Strategies for traditional and hybrid CME formats are also explored. Readers and institutions interested in developing CME, especially in the setting of the ongoing pandemic, will be able to use these strategies as a solid framework for producing CME. The recommendations and strategies presented within this paper are based on the authors’ opinions, expert opinions, and experiences over 13 years of creating CME events and challenges brought about due to the COVID-19 pandemic.

## Introduction

Continuing medical education (CME) is a requirement for medical doctors and various other health care professionals to remain competent within their respective fields [1-3]. CME is most effective when it is interactive, involves multiple exposures, and focuses on topics that clinicians view as important and timely [4]. When properly delivered, CME offers numerous benefits, including enhancing clinician knowledge, skills, and attitudes, improving patient outcomes, and lowering health care costs [5-10].

For many health care professionals, the COVID-19 pandemic upended many aspects of daily life, including professional obligations and the process of obtaining and delivering CME. The dynamic nature of the pandemic produced numerous challenges to the status quo of health care (clinical, education, and research) obligations. Given the travel restrictions, need for social distancing, and fear of “super-spreader events,” many in-person live CME courses were canceled outright across the country. In 2020, a study of CME planners demonstrated that 87% canceled their events due to the pandemic [11]

As the COVID-19 pandemic progressed to an endemic state, it became clear that given the ongoing need to fulfill professional licensure requirements, changes were necessary in the delivery of CME. The uncertainty of the pandemic and constantly changing policies catalyzed a need for flexible, on-demand continuing education for health care professionals, while still ensuring participant safety [12]. As a result, some CME events reemerged offering nontraditional formats, while others were eliminated entirely (Table 1) [13].

**Table 1:** Potential course formats [13-16].

Course format	Advantages	Challenges
Web-based course (enduring materials)	Learners complete at their pace Continuous revenue stream Flexibility for learner and expert	Accreditation requirements Content only valid as of date of recording Gauging audience response to content
In-person course	Networking with learners Gauging audience response	Cost Space limitations
Livestream course	Reduced travel costs Flexibility for speakers and learners	Technology issues and costs Professional image, that is, quality of the stream, branding Gauging audience response to content is more difficult without camera on livestream learners
Hybrid course (synchronous in-person and livestream)	Appeal to multiple learning styles Networking with learners Interaction with some learners during presentation Gauging audience response to content with learners in-person	Technology issues and costs Professional image, that is, quality of the stream, branding Engaging dual audiences Livestream audience has a feeling of being “left out”

Despite the importance of CME and the ongoing need for education, there is limited information on how to develop CME in the setting of the postpandemic era which includes many traditional and new formats such as conferences, workshops, enduring materials, or web-based and hybrid courses [14,15,17]. To close this gap, this paper puts forth an approach for creating and administering a CME event, including best practices for designing, implementing, and evaluating CME. Given that courses or conferences are the most popular format of CME, recommended strategies for conference-based traditional and hybrid formats will be reviewed, using published evidence and expert consensus informed by the new practices impulsed by the COVID-19 pandemic [14].

## Before You Create a CME Course

### Foundational Aspects of Collaboration and Communication

For a CME event to be successful, appropriate stakeholders must be identified [18]. Stakeholder support can vary greatly, from time, resources, expertise, mentorship, program and topic development, administrative assistance, and financial investment. CME requires a collaborative effort, supported by health care professionals, content experts, speakers, nonclinical support, and divisional or departmental or institutional leadership [19]. By incorporating other individuals with a shared passion and different skill sets, one will be able to efficiently strengthen the event across all stages.

Collaboration and open communication are fundamental factors throughout all stages of the CME. Course directors and speakers will need allocation of time away from their usual clinical practices to develop and take part in the event. Nonclinician support (program manager, CME specialist, public relations specialist, audiovisual experts, and other administrative staff) will also be needed. Depending on the size of one’s institution and the proposed CME, many of these roles could be combined. However, though it is possible to hire an external audiovisual support (either privately or from the destination property itself), using audiovisual support from one’s own institution and engaging them early in the planning process will ensure a successful event and cost control. Engaging with CME or event associations, such as the Alliance for Continuing Education in the Health Professions (ACEHP or Alliance), can provide resources to ensure compliance, templates, and staying up-to-date with best practices.

Institutional support and collaboration can be further secured by aligning the educational endeavor with one’s institutional needs [20]. Strategic needs can be measured using hard or soft data [20]. Hard data include financial and attendance goals, ability to meet educational objectives as measured via various evaluation methods, or clinical referrals to one’s institution. Soft data include the perceptions and opinions of the attendees gathered by word of mouth, open-ended comment sections on a standard evaluation, and anecdotal observations. By properly aligning the CME with the needs of one’s institution, one will be able to demonstrate the importance of the CME and the potential return on investment for the endeavor [21].

### General Needs Assessment

Once you have the foundational support, determine whether or not the proposed event or course is necessary or will add anything to the current educational landscape. The majority of successful CME come directly from the needs of those on the front lines of health care due to challenging cases or conditions, newly released guidelines, significant clinical practice changes, or the emergence of a novel subject [22]. As such, the proposed idea should be focused on, imparting new information or challenging previous knowledge, of interest to health care professionals, and impactful to the direct care of patients [22].

### Knowing Your Audience

#### Intended Audience

Identifying the intended audience is key to developing an effective CME. The planned audience will influence all aspects of the educational activity, from modalities to property location and credit types offered. Knowing the audience in conjunction with the learning gap, will also allow one to be able to formulate various aspects of the CME; this includes learning objectives, course content, marketing strategies, overall goals of the event, and preferred learning styles. A well-tailored instructional design is paramount as a recent study of CME preferences demonstrated significant differences in learning style preferences based on gender and medical specialty [23]. These variations in learning styles emphasize the recommendation to combine different educational modalities in a single CME event, which would allow greater educational flexibility for the attendees and better meet the needs of the audience related to the learning objectives [23,24]. It is worth noting, there is controversy regarding the validity of using learning styles when designing adult education; further research is needed to further explore the merits and limitations of learning styles [25].

Predicting the intended audience will also help in selecting the ideal location and venue destination for the CME. Health care professionals choose to attend a conference for multiple reasons, often traveling with nonconference participants (that is, family or friends). Thus, selecting a destination that has a distinguishable name brand and providing attractive activities for the nonconference participants, can positively impact course registrations. When selecting the location and creating the event program schedule it is crucial to consider the ease of access of the area (flight itineraries and ground transportation) and the local attractions and amenities that the venue and the surrounding area offer to participants and nonparticipants.

#### Competitive Assessment

When creating CME, one must explore the current educational marketplace [26]. To achieve this, perform a competitive assessment; a competitive (“needs”) assessment is a strategic tool, exploring the current state of the marketplace (that is, competition) [26]. A competitive assessment will help to identify the types of consumers that would most likely be interested, what the perceived positive traits in CME offerings are, and the strengths and weaknesses of the current competition. By researching the competition, one will be better able to differentiate the proposed event from the others, by focusing on specific details or gaps that are present in the marketplace.

The key to performing a competitive assessment is to identify what specific and actionable questions need to be answered [27]. By asking the “right” questions, a competitive assessment will also highlight the preferred modalities of the educational offering, associated operating costs, support or funding opportunities, and current industry standards for pricing of the CME.

Most of the information for the competitive assessment can be obtained from analyzing previous similar course programs, web-based searches, or by using companies that coordinate CME offerings [27]. Once the necessary information has been gathered, create an easy-to-read report that highlights the findings. The report should also provide actionable recommendations and foreseeable challenges. The competitive assessment should be updated (at least annually) in order to continually innovate the CME, mitigate risk, and be aware of changes in the competition [27].

#### Sourcing Properties and Event Timing

Determining the actual location and timing of the CME depends on accurately predicting the intended audience. By knowing the target audience, you will be in a far better position to select preferred destinations, timing options, program topics, and educational credit types that would attract a wide array of attendees.

While reviewing the competitive assessment, attention should be given to the timing of the proposed CME, the timing of similar educational offerings, and the timing of holidays. It is best to avoid offering a CME event during the week of a national holiday, spring break, or faith observance, as well as to keep several weeks of buffering between the proposed CME and related well-established, high impact CME offerings (such as national meetings).

In a recent survey, CME attendees indicated that they preferred to have courses during the work week, in the mornings, rather than during the weekend (personal communication by ABM, MD, Mayo Clinic Updates in Internal Medicine attendee survey, October 2020). As a result, the day of the week, time of day, and time zones should all be considered when determining the timing of the CME. Time zones are also especially important when incorporating a digital component to an event; a significant time zone difference can deter learners from attending hybrid, synchronous (simultaneous in-person and digital offerings) events that start early or end late in the digital learner’s time zone. Incorporating asynchronous (previously recorded) content would permit learners to review content at their own pace [28].

In terms of venue sourcing, this can be done by either contacting properties (hotels, resort, and conference centers) directly or using a property sourcing company. Textbox 1 outlines the key factors when sourcing properties.

**Key factors when sourcing properties.**

**Access**

- Closest large airport
- Car rental
- Additional lodging in area
- Additional activities for nonconference time
- Desirable location or weather

**Event space**

- Space to accommodate fluctuating audience size
- Proximity of breakout and meal rooms
- Proximity to guest rooms
- Food and beverage space

**Hotel and guest rooms**

- Ratio of guest rooms sufficient for event space
- Complimentary Wi-Fi
- Complimentary or reduced self-parking
- Waive resort fees
- Number of bathrooms in or near event space

**Audio or visual**

- Contract with hotel
- Reduced Wi-Fi expense
- Bring own equipment and technologists if possible
- Plan set up time with hotel at least 1 day in advance
- Plan clean up time at least 4 hours before next event set up begins

By using a sourcing company, one would be able to capitalize on any available resources and preexisting industry relationships that the sourcing company has with different sites or brands as well as bulk-price negotiation. Leveraging their expertise within the industry will help one negotiate lower rates and determine ideal properties and destinations best suited for the intended audience. Sourcing companies will provide one with a report enabling a quick comparison across multiple properties, brands, destinations, and dates. Another benefit to using a sourcing company is that in the event there are issues onsite, the sourcing company is able to intervene and help resolve issues promptly.

## Creating a CME Course

### Focused Needs Assessment

#### Course Objectives

Creating course objectives is the next step in developing a CME. By using the information from your general needs and competitive assessment, one should formulate the overarching goals of the CME program and objectives. Objectives should be specific, actionable, measurable, and relatable to the intended audience. It is recommended to create simple objectives, typically only 1 learning verb followed by a discrete knowledge or skills that springs from the gap analysis [22]. When writing measurable objectives, clearly indicate how the CME will measure the change in knowledge, behavior, or attitude.

#### Accreditation

While planning a CME course, one needs to consider the accreditation agencies specific to the target audience. Health care professionals are governed by different licensure board agencies, based on the respective specialty, which can have vastly different accreditation requirements and costs (Table 2) [22,29-32].

In addition, each state could have different regulations, guidelines, and CME requirements. To combat this variability, many of the specialty boards are now organized under the joint accreditation system of Accreditation Council for Continuing Medical Education (ACCME), which helps to streamline regulatory and accreditation procedures for interprofessional education [33]. All accrediting agencies require formulating course objectives, as well as educational and practice gap statements [29-32]. The educational gap statement highlights the overall educational need for the content being delivered. The practice gap is designed to describe and compare what *is* currently being practiced with what *should* be practiced. Clearly written educational and practice gaps are designed to guide the program development to achieve the documented objectives [22]. One should create this foundational component of the course, identifying what is the problem that the education offerings want to address, why this problem exists and how the education will help fill this gap.

The decision of which types of credit to offer is entirely dependent upon the intended audience. A course that offers multiple credit types (such as American Medical Association, American Board of Internal Medicine Maintenance of Certification, and American Academy of Family Physicians) can attract various subspecialties to the same CME.

**Table 2:** Commonly used United States accreditation bodies and credit type.

Name of organization	Credit
Accreditation Council for Continuing Medical Education (ACCME)	AMA^a^ PRA^b^ Category 1 Credit
AMA	AMA PRA Category 1 Credit
American Board of Internal Medicine (ABIM)	Maintenance of Certification (MOC) points
American Academy of Family Physicians (AAFP)	Family medicine
American Osteopathic Association (AOA)	Doctor of osteopathy
American Nurses Credentialing Center (ANCC)	Nurse or nurse practitioner
American Academy of Physician Assistant (AAPA)	Physician assistant
American Association of Nurse Practitioners (AANP)	Nurse practitioner

^a^AMA: American Medical Association.

^b^PRA: Physician Recognition Award.

### CME Content Development

#### Selecting Topics to Meet Your Objectives

The educational program is based on the overall purpose of the event, specific learning objectives, and clinical knowledge or practice gaps. The topics must be innovative, timely, and fill an educational gap that currently exists [34]. Using an iterative process with multiple stakeholders (course directors, CME administrators, and content experts) present, create a comprehensive list of potential educational topics and then discuss the benefits and limitations of each. This is particularly important while creating education for an interdisciplinary multispecialty audience. As the individual merits of each topic are assessed, ensure that each fulfills the course objectives and fits into the larger construct of the educational purpose and offering [22]. Topics that align with one’s objectives can be grouped together in a logical order, which will help to form a cohesive educational itinerary or program. Topics that do not meet these thresholds should be eliminated from the program.

#### Speaker Selection

Once the course objectives and educational topics are finalized, move on to speaker selection. Many factors are involved with speaker selection, including identification of content experts, content delivery skills, inclusion and diversity, institutional priorities, and scheduling availabilities [35]. Be mindful of any gender or racial disparities in the speaker roster. Furthermore, sending an announcement to gauge the level of interest that individuals at your institution might have for future speaking opportunities can lead to additional potential speakers and content experts. Faculty evaluations completed by the course participants can also assist in determining future speakers that resonate with the attendees.

### Planning the CME

#### Budget

CME is an expensive undertaking with a very large overhead. Upfront resource identification and allocation is needed for developing and operationalizing the educational event. Many expenses (including deposits, marketing strategy, and printing educational materials) are incurred prior to the event. However, the 2 largest expenses, food or beverage and audiovisual, are not incurred until the CME begins [36]. By comparing data from previous events with similar audience demographics, one can create a realistic budget and effectively manage expenses. In fact, the initial step to position a course for successful budget management occurs during the competitive assessment. It is during the competitive assessment that resources such as CME planning staff, audio visual staff, accreditation fees, marketing fees, and clinical course leadership time should be assessed.

The post–COVID-19 era is characterized by more diverse types of educational offerings, with web-based and hybrid CME events becoming much more common [37]. Budgeting for a web-based or hybrid synchronous event should include the cost of the additional technology necessary to successfully deliver the content digitally. Initially, there will be some upfront additional expenses, given that many CME courses have never had a web-based presence. After the initial purchase of the equipment, ongoing expenses will include continued maintenance and operation of the equipment and upgrading the technology as needed. Though there exists a perception among attendees that web-based events should be offered at a lower cost due to the reduction of travel and food, a high-quality web-based or hybrid CME will require greater resources and technology, which will ultimately drive up the costs of producing the CME [38]. It is important to develop and provide a high production value for hybrid or live streaming educational events.

#### Marketing

Marketing strategies should be reviewed throughout the life cycle of the activity. Marketing expenses can be one of the more expensive aspects of hosting a CME event [36]. Types of marketing include email, direct mail, website, paid search, web-based calendar, print and web-based journals, industry specific organizations, and social media. Careful consideration of the intended audience will help to identify the most effective marketing strategies. The intended audience’s marketing preferences can differ based on the specific course and audience demographics. According to a recent physician survey, 84% of physicians report email and direct mail as the top 2 marketing techniques leading to course registration [39].

The above survey also noted that physicians received marketing information 2-3 times before deciding to attend a CME event [39]. This highlights the need to develop a multiphase, multimodal media (that is, email, web links, brochure, and social media) strategy. When deploying a multimodal marketing campaign, use similar messaging in the different marketing approaches [40]. Further, word-of-mouth marketing can be influential. Word-of-mouth marketing can be face-to-face or via social media. A recent study demonstrated that word-of-mouth via social media was more influential than direct face-to-face communication [41].

Marketing strategies should also be tailored, based on generational demographics of the target audience [42]. In general, there are 3 generational demographics that most likely attend CME events: baby boomers (born 1955-1964), Gen X (born 1965-1976), and millennials (born 1977-1992) [43]. Understanding generational similarities, differences, and preferences can guide the marketing strategies. According to a 2018 study, millennials prefer email (77%) and web-based searches (65%) as sources for event information, while Gen X prefer print media via direct mail (65%), followed by email (59%), and web-based searches (59%) [44]. Though millennials are most likely to engage in social media sites, digital activity by Gen X and baby boomers continues to grow [44]; thus, it is important to target these groups digitally as well. Furthermore, while Gen X is a smaller population compared to millennials or baby boomers, they actually have the largest CME-related spending power, making it a highly sought-after demographic group [44].

In order to track this information, one should implement conversion tracking for digital marketing tactics. Conversion tracking identifies how well an advertising campaign is performing by tracking web-based traffic from digital marketing that leads to actual registrations. Furthermore, it is recommended to ask how the attendee learned about the event during the registration process; this information can then be used to guide future marketing strategies [39].

#### Course Website

**Figure 1:**
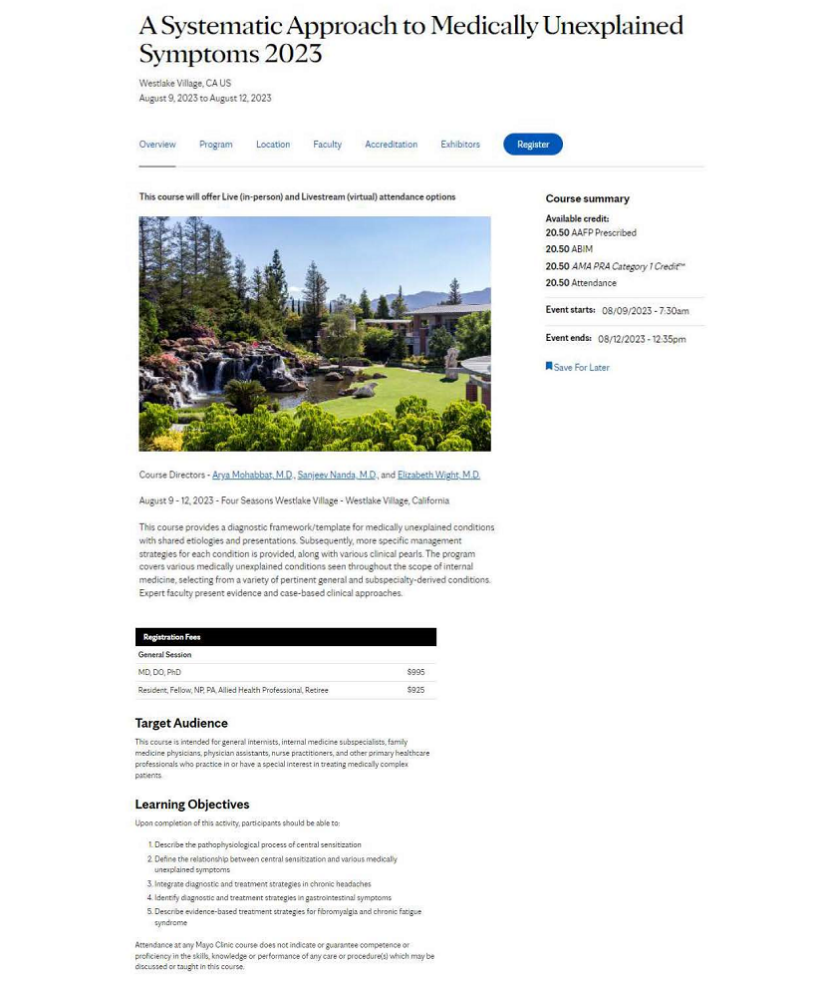
Example of course website layout.

The overall goal of your marketing campaign is to increase registrations. Thus, the course website and web-based registration platform are an integral part of the marketing campaign. The website will function as a centralized space from marketing, to registration, through claiming credit. Figure 1 is an example of the layout of a course website. Textbox 2 offers various effective suggestions when creating a CME course website [45].

Disorganized or incomplete websites reflect poorly on the course and could deter attendees from registering. The participant’s interaction with the course website can influence the overall perception and success of the course as it would be the first (general information and registration) and last (evaluation and credit claiming) impression an individual would have with the CME [45]. Multiple vendors exist in the domain of website development and administration for CME activities, however it is essential for the organizers and owners of the content to keep close supervision of the branding and functionality of the platform.

**Strategies for creating course website.**

**Website**

- Clearly state value proposition of course
- Easy to navigate
- Mobile friendly
- Internet search results link directly to desired information
- Organic results appear after an internet search has been initiated
- Based on “ranking” (relevancy of search term to course listing)
- Enhance using search engine optimization (SEO)

**Details needed**

- Location
- Description
- Start and end times (include time zone)
- Program schedule
- Registration platform
- All course materials
- Syllabus
- Speaker list
- Digital delivery details and links (as needed)
- Accreditation requirements
- Claiming credit

**Search engine optimization (SEO)**

- Consistent web address with multiple pages
- Static (unchanging) pages
- Specific year-to-year course changes
- Meta title—appear as the results of internet search
- Concise
- Meta description—appear under the meta title
- Clearly describe the value of the course
- Keywork targeting
- Terms potential audience is using to locate desired event
- Strategic and unique to course

**Digital delivery tips**

- Consistent across portfolio
- Software for digital delivery
- Consistent access point
- Flexible
- Tested prior to event
- Available throughout the event
- Enhance digital user experience
- High quality production value
- Responsive video platform
- Provide opportunity to interact with speakers and be engaged

#### Commercial Support

Commercial support is often sought to offset the expense of planning, marketing, and running the CME. Commercial support can be in several forms, including grants, scholarships, vendor displays, sponsored events, or in-kind contributions. Each commercial entity has their own application process to apply for funds. Leveraging established relationships and contact points between one’s institution and industry could significantly facilitate this process.

This expense mitigation however comes with an implicit appearance of commercial bias and conflicts of interest [46]. To prevent overt bias or conflicts, all team members should follow the ACCME Standards for Integrity and Independence in Accredited Continuing Education policies, that put forth strong regulations when involving commercial support [47]. Compliance with these policies and formal documentation of mitigating plans are required by all accrediting agencies. Additionally, policies under the 2010 “Sunshine” provisions require extensive disclosure reporting for speakers, event planners, authors, and other participants; all relationships and conflicts of interest must be documented, made available, and resolved prior to the educational event [48].

## Running and Evaluating the CME

### Onsite Strategies

The CME organizers should build a positive operating relationship with the venue and technology managers in order to ensure a professionally delivered experience. This relationship begins with clear communication of the needs and expectations and what they in turn can expect from the CME organizers [49]. These communications will usually occur over a period of months, in the form of phone calls, emails, digital platforms, and potentially a pre-event onsite visit. Once at the venue, a preconference meeting should be scheduled with the venue managers; this allows for an opportunity to clarify any questions about the program and audience, verify safety and security measures, and review the layout of the hotel and conference space areas. For a web-based event, the technology and CME organizers should review the electronic set up, identifying the support structure for the attendees, and discuss strategies to minimize or resolve any potential technological issues.

For an onsite event, the setup of the conference room reflects on both the CME organizers and the venue. Each event will need multiple separate spaces, each serving its own purpose [49]. Specific areas will be needed for the registration desk, networking, formal educational sessions, audiovisual or technical support, refreshments, and commercial support. Room set up should allow for smooth traffic flow and allow for easy access to refreshments, restrooms, and the registration desk, all while providing for appropriate social distancing. Regulations by ACCME forbid the coexistence of educational and commercial activities in the same physical space unless they are separated by a considerable amount of time. Conference materials should be made available for quick access by attendees and flow directly into the educational space or have them easily available from a web-based depository.

The refreshment area should be in a location close to, but separate, from the educational space; this will allow for a more relaxed environment for breaks, discussions, and networking, while not disrupting the educational offerings. When designing the seating arrangement in the educational space, select a room set up that encourages the goals of the event. Classroom style seating provides space to use electronic devices and note taking; crescent or full round tables encourage engagement between attendees; theater style maximizes space to accommodate large groups [50]. When determining the layout, identify if tablespace is needed for audience note-taking, the need for charging outlets, or the ability to stand during the learning sessions.

Pandemic-related health and safety precautions will vary based on the local governance and will impact the layout of the educational space. The Centers for Disease Control’s recommendation for social distancing of 6 feet has greatly reduced the capacity and layout of the learning environment [51]. It should also be noted that some institutions and venues will have their own policies. In addition, social distancing creates a challenging situation for professional networking. Conference sites may also recommend numerous modifications to their facilities to limit the amount of people passing through specific areas. These precautions will impact the flow, timing, and set up of the event. As a result, additional noneducational (“break”) time will need to be built into the schedule to allow for appropriate distancing, while still fostering a welcoming and collegial environment. Despite the recent normalization of regulations around in-person events, it is prudent to expect unforeseen medical and social events that can rapidly change policies and impact the operations around the educational offering.

Opportunities for direct communication between attendees and CME representatives (course directors, speakers, and administrators) should be carefully reviewed. The CME organizers will need to provide customer support and be readily available to resolve questions or concerns throughout the lifecycle of the CME. This is true both for in-person and web-based events. In total, 1 event member should be at the registration desk at all times at a destination event to provide customer support for the attendees and serve as a security measure for the event materials. Furthermore, course directors and faculty members should be located in the educational space, moderating the conference, communicating any changes in the program or timing to the organizers and technology staff, and facilitating introductions and question and answer sessions. Close communication will provide for seamless transitions between sessions, facilitate any timing adjustments, and resolve any urgent issues. The advent of web-based–only and hybrid events creates a clear need to allocate enough resources, often a designated administrative staff, focused on the needs and particularities of the digital offerings. Issues with livestreaming, web-based troubleshooting, accessibility, and overall good-customer support mandate that as much attention be paid to the in-person customers as well as to digital ones.

### Course Evaluations and Outcomes

What constitutes whether an event has been successful or not should be determined during the initial planning stages and documented, allowing you to directly compare the event’s performance to specific objective and subjective metrics. Performance metrics could include registrations, attendance, financials, marketing, hotel block occupancy, institutional referrals, and course evaluations.

Course evaluations are more subjective than the other performance metrics mentioned. However, all attendees should have the opportunity to evaluate the course, assessing its impact on their knowledge gaps and patient outcomes. Course evaluations can be created using different formats, depending on what is being measured [22]. Surveys, questionnaires, pre- or posttests, and reflective questions are just a few examples [22]. The course evaluation should capture the satisfaction of the learning event, indicate if course objectives were met, and reporting of likely changes to their practice due to the CME [52]. The course evaluation can assess not only the impact on the clinician to remain competent within their respective field, but could also be used to solicit recommendations for future topics or locations. All evaluations and suggestions should be combined into a report and reviewed by the CME organizers. As previously mentioned, postactivity evaluations are very important to inform the effectiveness of the education, the achievement of the learning objectives, and to provide several data points to appraise the success of the course or web-based activity.

## Summary

### Principal Findings

CME is a requirement for health care professionals to stay current in their ever-changing fields. The COVID-19 pandemic significantly impacted the process of creating and administering CME. To successfully provide CME in the post–COVID-19 pandemic era, an institution and its programs must not only understand their audience and educational goals, but more importantly, appreciate and use the novel formats and delivery platforms. By doing so, one will be able to determine the most appropriate modality, timing, location, and marketing strategies. A CME event requires collaboration between stakeholders, accreditation agencies, and commercial support; clear communication and realistic expectations are vital when developing CME. Given the post pandemic-related challenges, CME providers are faced with significant uncertainties moving forward. The long-term impact of this transition on learning preferences and knowledge retention has yet to be determined. Nevertheless, given these changes, institutions will need to quickly adapt on how best to encourage ongoing CME participation and improve both the web-based and traditional destination experience for their attendees.

This viewpoint paper does have several limitations that should be highlighted. First, the strategies are based on a combination of author and expert opinion, as well as our collective experience with CME development and administration. As such, there can be selection and recall bias, as well as limitations in generalizability. Second, our institution is a large tertiary academic medical center based in the United States; this could limit the generalizability and applicability of our recommendations. Third, it should also be noted that there is increasing discussion regarding the utility and validity of adult learning style or theory and its application in CME. We acknowledge this dispute, but also note that our institution does rely and use adult learning theory principles in CME development.

## Abbreviations

ACCME: Accreditation Council for Continuing Medical Education
ACEHP: Alliance for Continuing Education in the Health Professions
CME: continuing medical education

## Data Availability

All data generated or analyzed during this study are included in this published paper [and its supplementary information files].
